# Proton‐Transfer Ferroelectrics With High Switching Endurance

**DOI:** 10.1002/smsc.70438

**Published:** 2026-11-05

**Authors:** Bibek Tiwari, Yuanyuan Ni, Xiaoshan Xu

**Affiliations:** ^1^ Department of Physics and Astronomy University of Nebraska‐Lincoln Lincoln Nebraska USA; ^2^ Department of Physics and Astronomy and the Nebraska Center for Materials and Nanoscience University of Nebraska‐Lincoln Lincoln Nebraska USA

**Keywords:** crystallinity, crystallization, ferroelectricity, hydrogen bonded network, materials science, molecular physics, organic compound, temperature cycling

## Abstract

Reliable organic ferroelectrics for memory applications require high endurance under repeated electrical switching. Here we demonstrate robust fatigue resistance in highly crystalline 2‐methylbenzimidazole (MBI) films grown by low‐temperature deposition followed by restrained crystallization (LDRC) on interdigital electrodes. Switching kinetics analyzed using the Kolmogorov–Avrami–Ishibashi (KAI) model reveals characteristic millisecond switching times and quasi‐one‐dimensional domain growth associated with proton transfer along hydrogen‐bond chains. Guided by these kinetics, we examined the switching endurance under a stringent protocol that ensures complete polarization reversal. After ~10^8^ continuous cycles (~2 weeks), the remanent polarization shows a small initial wake‐up behavior (~10% increase within the first 10^4^ cycles), followed by stabilization at values close to the initial polarization, indicating robust ferroelectric endurance under prolonged electrical cycling. The combination of LDRC‐enabled high crystallinity and localized proton‐transfer switching, which introduces minimal structural perturbation during polarization reversal, may have enabled this fatigue tolerance and highlight MBI as a simple, fluorine‐free platform for durable organic ferroelectric devices.

## Graphical abstract

Stable polarization retention over high‑field switching cycles is demonstrated in LDRC‑grown hydrogen‑bonded 2‑methylbenzimidazole (MBI) thin films, revealing reliable ferroelectric endurance. The inset presents the two‑layer molecular structure with dotted hydrogen‑bond pathways, providing the structural basis for the observed long‑term switching stability.

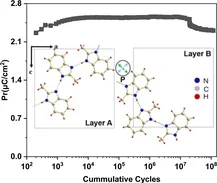

## 1 Introduction

Reliable organic ferroelectrics are essential for flexible nonvolatile memories, sensors, and actuators [1, 2], where devices must sustain large numbers of switching events without significant polarization loss. Polymer ferroelectrics such as PVDF and P(VDF–TrFE) remain the most widely studied organic systems, offering sizable polarization and convenient solution processability [3]. However, their long‑term reliability is limited by polarization fatigue, which is strongly influenced by interfacial charge injection, charge trapping, and field‑driven transport processes [4]. In fluorinated polymers, electron‐induced C─F bond breaking can produce HF that accumulates in the device stack, leading to interfacial degradation, electrode delamination, and reduced switchable polarization [3,5, 7]. Although optimized electrodes, dielectric interlayers, and tailored driving schemes have improved endurance, fatigue in polymer ferroelectrics remains closely tied to device architecture and interfacial chemistry [8].

These considerations have motivated increasing interest in molecular ferroelectrics, where polarization arises from well‐defined intermolecular interactions rather than polymer chain conformations [9]. Hydrogen‐bonded molecular crystals, such as polycarboxylic acids and benzimidazole derivatives, are particularly promising, as their ferroelectricity originates from proton transfer along ordered hydrogen‐bond networks, enabling polarization reversal through localized proton motion rather than large‐scale structural rearrangements [9, 10, 11, 12, 13, 14]. This localized proton‐transfer mechanism can, in principle, reduce structural disruption per switching event and thereby improve fatigue endurance, especially when combined with high‐crystallinity films that minimize defect‐mediated pinning. Yet, systematic fatigue studies on molecular proton‐transfer ferroelectrics and their microscopic switching kinetics remain scarce.

Here, we investigate ferroelectric switching and long‐cycle endurance in films of highly crystalline 2‐methylbenzimidazole (MBI), a hydrogen‑bonded molecular ferroelectric in which polarization reversal occurs via proton transfer along N─H···N bonds [15, 16, 17] (Figure 1a), grown by a low‐temperature deposition followed by restricted crystallization (LDRC) process on interdigital Pt electrodes prepatterned on glass. We show that LDRC produces spherulitic films with micrometer‐scale fibers and near single‐crystal‐level polarization. The polarization switching measured on the interdigital structure reveals Kolmogorov–Avrami–Ishibashi (KAI)‐type switching [18] and with Avrami index *n *≈ 1 and a Merz’s law [19]–type dependence on the applied electric field, consistent with nucleation‐dominated switching mechanisms. Building on this kinetic understanding, we design a stringent high‐field fatigue protocol and demonstrate that MBI maintains its remanent polarization closer to the initial value after 10^8^ cycles, corresponding to ≈2 weeks of continuous high‐field operation, highlighting robust fatigue resistance compared with conventional P(VDF‐TrFE)‐based ferroelectrics.

**FIGURE 1:**
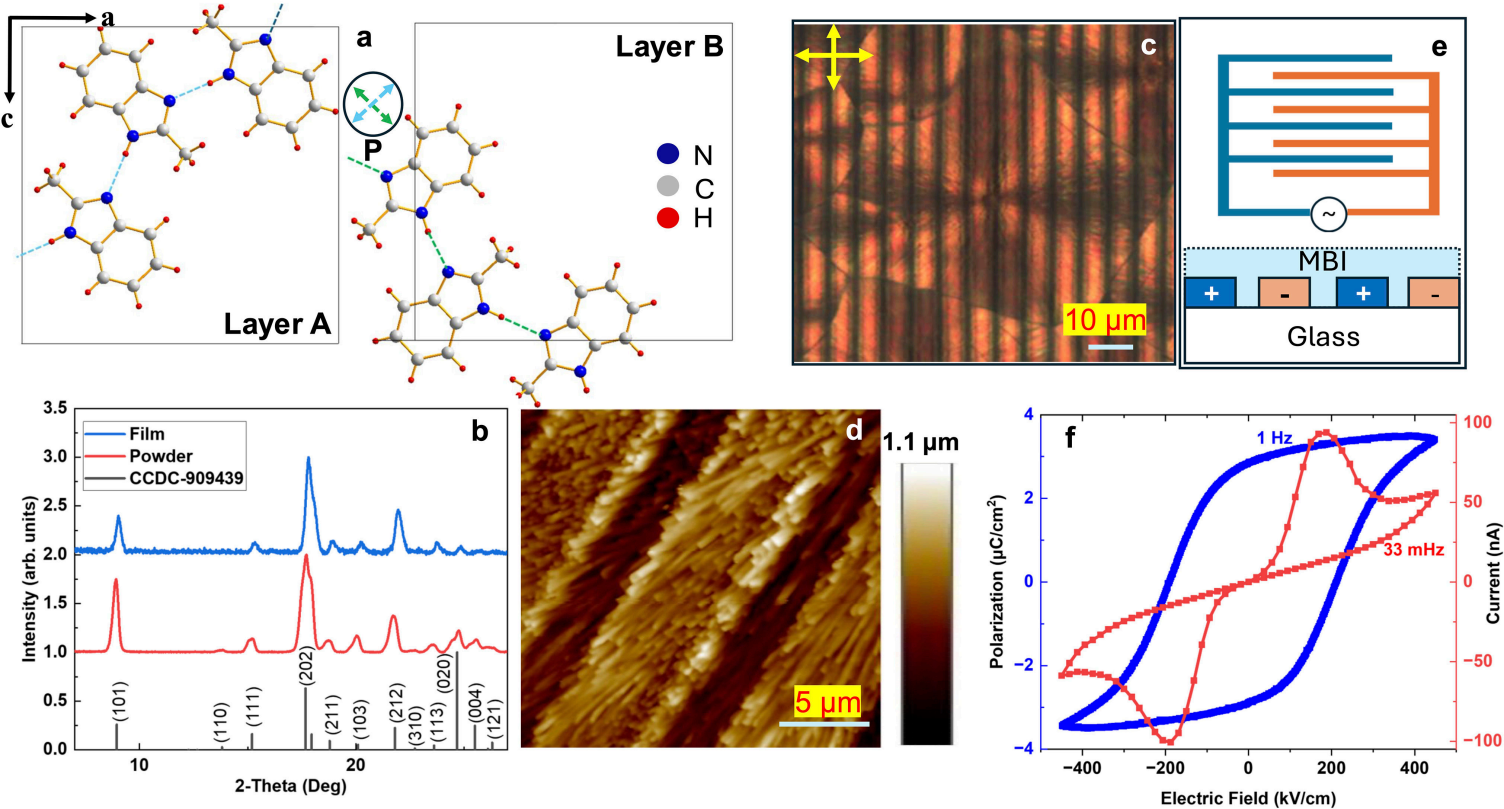
Structural origin, microstructure, and ferroelectric response of the molecular MBI organic film. (a) Polar crystal structure highlighting hydrogen‐bonded molecular layers and proton‐transfer‐induced spontaneous polarization (shown by green and blue arrows and dots, polarization direction shown inside red circle). (b) X‐ray diffraction patterns of the thin film compared with powder and reference data, confirming phase purity and preferred orientation. (c) Crosspolarized optical microscopy image showing anisotropic ferroelectric domain morphology and maltese cross patterns. The stripe patterns are from the interdigital electrodes (see panel e). (d) Atomic force microscopy topography revealing fiber‐like crystallites. (e) Schematic of the interdigitated electrode (IDE) with gap size of 4 μm and period of 10 μm configuration used for in‐plane electrical measurements. (f) Polarization–electric field (*P–E*) hysteresis and current–electric field *(I* *–* *E* *)* loops measured under IDE geometry, demonstrating switchable ferroelectric polarization driven by proton transfer within hydrogen bonds. Film thickness is 7 μm.

## 2 High Crystallinity in LDRC Films

The MBI films examined in this study were deposited by physical vapor deposition directly onto an interdigitated electrode (IDE) on glass substrates. This was achieved using low‐temperature deposition followed by restrained crystallization (LDRC) method at 230 K, following the procedures reported in earlier studies [16, 20, 21]. The out‐of‐plane X‐ray diffraction (Figures 1b and S9) confirms that the thin film (7 and 2 μm) adopts the bulk polar monoclinic phase, with reflections matching the simulated and powder reference pattern (CCDC‐909439). Direct optical microscopy images in Figure S1a reveal spherulite‐like features with apparent nucleation centers. The spherulitic nature is confirmed by crosspolarized optical microscopy, as shown in Figure 1c and in Figures S1b and S8. Well‐defined maltese crosspatterns are observed within individual spherulites, evidencing radially oriented, optically birefringent crystalline structures characteristic of spherulitic growth. Complementary atomic force microscopy in Figure 1d further resolves μm‐sized rod‐like crystallites within the spherulite fibers, with ~500 nm diameters and >1 μm length.

Ferroelectric switching is probed using an interdigital electrode geometry, as schematically shown in Figure 1e. As shown in Figure 1a, MBI crystallizes in a bipolar ferroelectric structure composed of two molecular layers per unit cell; within the A and B layers the hydrogen‐bond chain defines the polar axis along the [$10\bar{1}$] and the [101] directions respectively. This makes the polarization possible along any direction within the *a*‐*c* plane. As shown in Figure 1f (PE loops for other frequencies and temperatures are shown in Figure S3), well‐defined *P–E* hysteresis along with I–E loop with clear remanent polarization (*P*_r_) and coercive fields is measured. The corresponding *P* *–* *E* and *I* *–* *E* loops for a 2 µm sample are shown in Figure S6. Notably, the *P*_r_ value is comparable to the single‐crystal values (*P*_r_ ≈ 5.2 μC/cm^2^ at 300 K, 0.2 Hz) [9, 11, 14, 16], consistent with the large crystallite size and bipolar nature of MBI. The slightly reduced *P*_r_ can be attributed to interdigital geometry of the sample structure.

Taken together, these results indicate that the LDRC process produces highly crystalline MBI films. The combination of well‐defined spherulitic morphology, large crystalline fibers, and near single‐crystal‐level ferroelectric polarization provides an excellent platform for investigating polarization switching dynamics and fatigue behavior.

## 3 Switching Dynamics and KAI Behavior

To determine the switchable polarization, measurements were performed using a ferroelectric tester following the pulse sequence shown schematically in Figure 2a. The first (positive) voltage pulse initializes the sample into a well‐defined polarization state. The second (negative) pulse, characterized by a pulse width (*t*_p_) and pulse voltage (*V*_p_), is applied to induce reverse polarization switching. A delay time Δ*t* = 100 ms was maintained between successive pulses to minimize transient effects. The third (positive) pulse is used to measure the polarization switch by the second pulse. However, this signal ($P^{*}$) contains contributions from nonferroelectric effects such as leakage current and capacitive charging. To account for these contributions, a fourth (positive) pulse is applied to record the nonswitchable polarization ($P^{\wedge }$). The net switchable polarization is then obtained as $P_{sw}=P^{*}-P$ for a given pulse voltage and pulse width.

**FIGURE 2:**
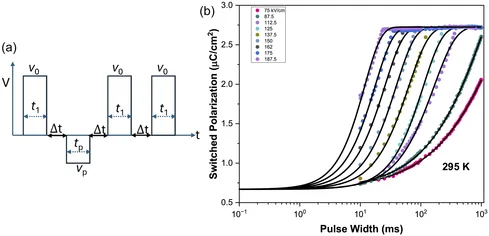
(a) The pulse sequence scheme for switching dynamics measurement with *V*_0_ = 190 V, Δ*t* = 100 ms, and *t*_1_ = 1000 ms, *V*_p_ and *t*_p_ are varied. (b) Switchable polarization (*P*_sw_) versus pulse width (*t*_p_) at 295 K for various electric fields *E* (by varying *V*_p_) and solid lines are fit to KAI model. Film thickness is 7 μm.

Figure 2b shows the switchable polarization (*P*_sw_) as a function of pulse‐width (*t*_p_) on a logarithmic timescale for several applied electric fields measured at room temperature (~295 K) for various electric fields. Clearly, increasing the applied voltage (electric field) leads to faster switching. For high electric field and long pulse widths, $P_{sw}$ saturates at ~2.8 µC/cm^2^. Below 112.5 kV cm^−1^, no clear saturation of polarization is observed up to 1000 ms, suggesting longer switching time. This behavior agrees with typical ferroelectric switching models in which stronger electric fields reduce the characteristic switching time by enhancing domain nucleation and domain wall motion [22, 23, 24, 25, 26, 27].

The experimental data were analyzed by fitting to the KAI model as given by Equation (1),

(1) $$\text{Δ}P_{sw}\left(t\right)=2P_{s}\left[1-\exp\left(-{\left(\frac{t}{t_{0}}\right)}^{n}\right)\right]$$

where *P*_s_ is the saturation polarization, *n* is the Avrami index, and $t_{0}$ is the characteristic switching time. The baseline value of polarization was estimated based on measurements with lowest electric field and shortest pulse width (10 ms, limited by the instrument) with extrapolation to lower time scale.

The agreement between the experimental results and the KAI model shown in Figure 2b indicates that the polarization switching in the MBI film is governed by nucleation‐and‐growth dynamics. The KAI theory was originally formulated for displacive inorganic ferroelectrics to describe an “ideal” case of switching with a narrow distribution of nucleation time and domain wall velocity. Ideal KAI behavior is typically restricted to large single crystals with spatially homogeneous switching; it is less seen in thin films due to contributions from interfaces, grain boundaries, and defects. Therefore, the excellent fit in Figure 3b suggests high crystallinity inside the large (μm‐sized) rod‐shaped crystallites (Figure 1d), indicating minimal contributions to the switching kinetics from the grain boundaries.

**FIGURE 3:**
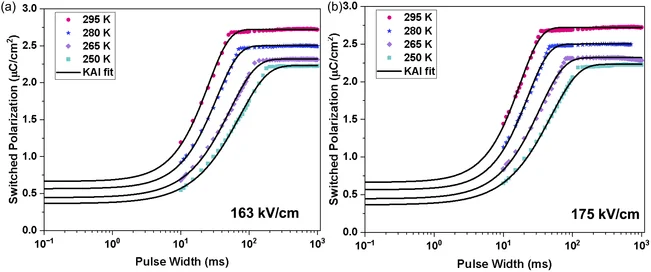
(a) Temperature dependence of switchable polarization at a fixed electric field of 163 kV/cm (several field dependences at a given temperature are shown in S4), illustrating slower switching at lower temperatures and solid line is a fit to KAI model. (b) Similar temperature dependence graph at a fixed electric field of 175 kV/cm. Film thickness is 7 μm.

## 4 Quasi‐1D Switching and Merz‐Type Kinetics

To understand the role of thermal activation in polarization switching, the dependence of the switchable polarization on pulse width was investigated over the temperature range of 250–295 K with similar electric field (see Figure S4 for detail $P_{sw}$ at several electric fields for each temperature). Figure 3 illustrates the temperature‐dependent polarization switching dynamics of the sample. At both electric fields in Figures 3a,b, an increase in temperature results in a systematic shift toward smaller switching time. This aligns with the thermally activated nature of domain nucleation and growth, where increased thermal energy yields a higher probability of overcoming the switching barrier, as observed in polymeric systems like PVDF [25].

The Avrami’s index *n* in Equation (1) serves as an indicator of the effective dimensionality of domain‐growth kinetics and the nature of the nucleation process; the extracted values are plotted in Figure 4a. At low temperature, the low‐field regime yields Avrami indices close to 1, consistent with predominantly one‐dimensional switching. At higher temperature or higher fields, n increases modestly, indicating that thermal fluctuation and strong electric fields activate a transition from predominantly one‐dimensional to slightly more multidimensional switching pathways. Intrinsically, *n* ≈ 1 reflects the anisotropy of proton‐transfer switching in MBI. As shown in Figure 1a, proton displacement occurs preferentially along H─bonded N─N···N chains, creating quasi 1D switching pathways at low fields. Lateral expansion of switchable regions requires correlated molecular reorientation across neighboring chains, which is energetically less favorable, thereby leading to domain growth effectively constrained to 1D at lower fields. The near‐unity values confirm that polarization switching tracks the hydrogen‐bond chains, another indication of high crystallinity, since abundance of defects may undermine the 1D behavior. At higher fields, additional nucleation sites become active for transverse nucleation, enabling a more extended growth geometry reflected in the rising n values. Conversely, n decreases systematically with decreasing temperature, indicating a suppression of nucleation activity and increasingly constrained domain‐wall motion as thermal energy is reduced.

**FIGURE 4:**
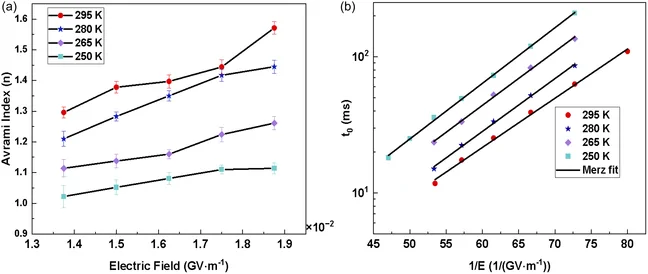
KAI model parameters. (a) Avrami index n as a function of electric field at the same temperature range, indicating field and temperature‐dependent domain‐growth kinetics. (b) Plots of characteristic switching time $t_{0}$ versus inverse electric field 1/E for 250–295 K, showing linear behavior consistent with Merz’s law (solid lines are fits). Film thickness is 7 μm.

The characteristic switching time $t_{0}$, extracted from the KAI model fit, is plotted in Figure 4b as a function of 1/*E* for all temperatures to examine its compliance with Merz’s empirical law,

(2) $$t_{0}=t_{\infty }\exp\left(\frac{\alpha }{E}\right)$$

where *α* is an activation field associated with the energy barrier for domain reversal and *t*_∞_ is the switching time at an infinite field. The linear behavior observed in the semilog plot confirms that the switching dynamics follow Merz‐type kinetics across the entire temperature range.

The nearly parallel lines for different temperatures indicate that the activation field α is only weakly dependent on temperature within this range. Quantitively, from the fits shown in Figure 4b, the extracted values of *t*_∞_ and *α* at 295 K are approximately 0.14 ms and 0.08 GV m^−1^ respectively. This slow switching time scale is also evident in BTA‐C18‐type systems [27], but the slow nature comes here with long carbon chains with higher masses favoring delayed switching unlike for BTA‐C6 (~µs). In MBI, the strong correlation between the proton position and the *π*‐electron states in the C─N rings could slow down the proton transfer in the collective macroscopic behavior [10]. In addition, this α could also be overestimated due to the IDE nature of in‐plane electric field measurement for order less than that observed in PVDF‐type polymeric systems [24, 27]. However, in contrast to PVDF systems, where switching involves conformational changes of polymer chains [28], MBI switching is driven by localized proton transfer within rigid molecular frameworks. This distinctive nature also might favor the lower activation fields in general.

Overall, upon lowering the temperature (Figure S5), both α and $t_{\infty }$ increase, although the temperature dependence of α is clearly weaker (Figure S5a). In proton‐transfer ferroelectrics, the activation field extracted from Merz law reflects the field required to distort/tilt proton double‐well within H‐bond. Unlike displacive ferroelectrics, where barrier is dominated by collective ionic motion, the barrier in MBI arises from localized proton transfer, the fundamental switching unit is a proton hopping between adjacent sites [10]. This localized nature of the switching unit is possibly weakly coupled to the lattice, which leads to a modest temperature dependence of the activation field, so a weaker temperature dependence of the activation field is plausible. On the other hand, the nucleation is inherently temperature dependent, as also discussed in TA‐NLS [29] model, causing the stronger temperature dependence of the characteristic switching time $t_{\infty }$.

## 5 Robust Fatigue Resistance

Leveraging our understanding of the switching kinetics, we designed a stringent testing protocol to evaluate the fatigue resistance of the MBI films. At room temperature, 2 μm‐thick MBI films show a similar polarization switching behavior to the 7 μm‐thick films (Figure S7a and Table S3), characterized by an Avrami index of *n* ~ 1 (Figure S7b) and switching time *t*_0_ slightly smaller than that of the 7 μm‐thick films under the same condition (temperature and electric field) (Figure S7c). We utilized a bipolar voltage pulse of 180 V—approaching the limit of the tester—which corresponds to approximately 450 kV/cm (~2 × *E*_c_). Based on the Merz law fits (Figure S7c), the characteristic switching time *t*_0_ at this specific field is estimated to be around 0.84 ms. Therefore, we selected a pulse width of 5 ms for fatigue cycling. This pulse duration ensures complete polarization reversal during every cycle, avoiding the partial‐switching artifacts, which may inflate cycle counts in high‐frequency endurance testing.

The fatigue measurements were performed on a 2 μm‐thick MBI film continuously for up to 10^8^ cycles (approximately 2 weeks) under these high‐field conditions. To account for nonferroelectric contributions such as leakage current, the switchable polarization was measured intermittently using the positive‐up‐negative‐down (PUND) method (see Supporting Information: Note S1).

As shown in Figure 5a, the remanent polarization (*P*_r_) initially increases and saturates after 10^3^ cycles. This is indicative of a “wake‐up” effect, a well‐known phenomenon driven by the field‐induced de‐pinning of domain walls, relaxation of internal stresses from the growth process, and the stabilization of interfacial charge screening. Following this initial wake‐up phase, the polarization remains remarkably constant up to ~10^7^ cycles, demonstrating excellent fatigue endurance.

**FIGURE 5:**
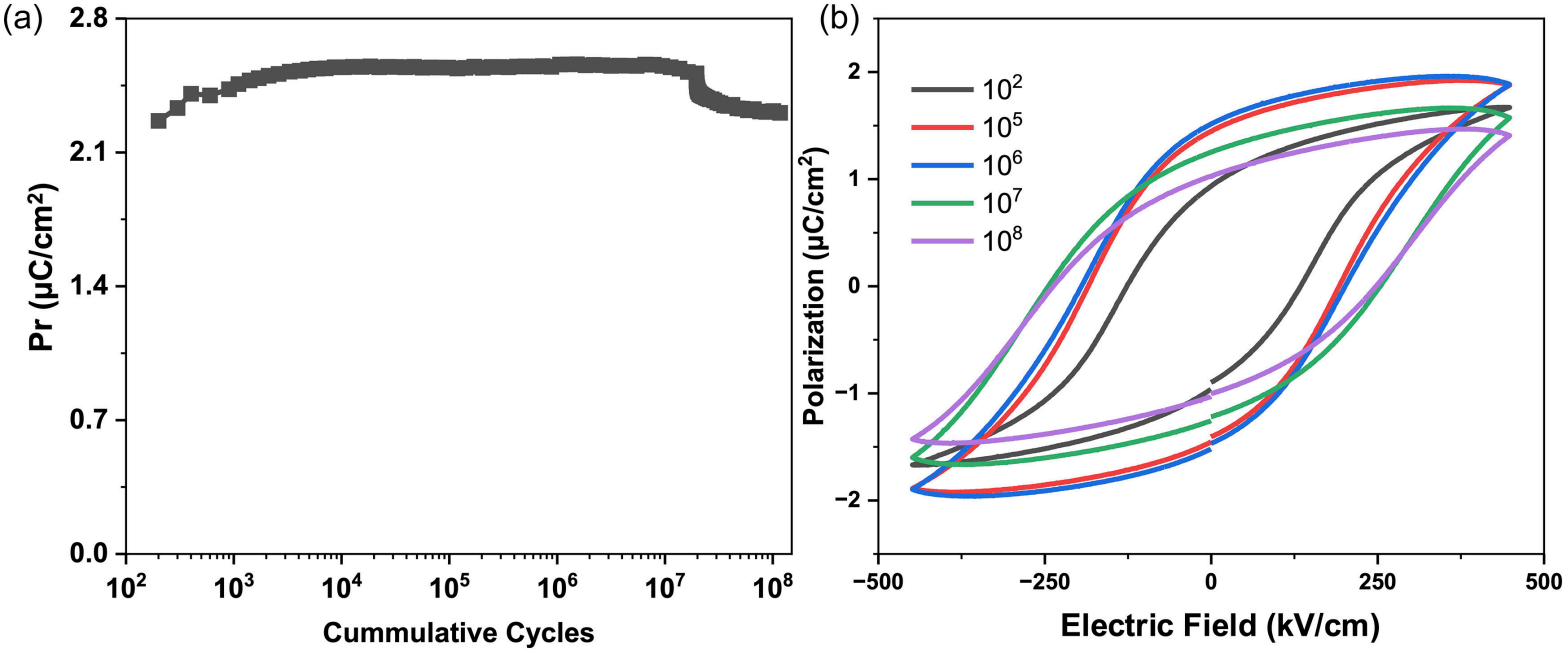
Fatigue behavior of the MBI ferroelectric film measured using bipolar pulses of 180 V amplitude and 5 ms width. (a) Remanent polarization $P_{r}$ as a function of cumulative switching cycles. (b) Corresponding *P–E* hysteresis loops at selected cycle numbers, showing the evolution of polarization switching during repeated high‐field cycling. Film thickness is 2 μm.

At higher cycle numbers, a slight degradation emerges; however, the overall reduction in *P*_r_ from its peak value remains below 10%. Ultimately, at 10^8^ cycles, the polarization stabilizes at a value nearly the same as the pristine, uncycled state. The corresponding *P* *–* *E* hysteresis loops in Figure 5b corroborate this trend, showing the initial increase followed by the gradual decrease of *P*_r_, accompanied by a consistent, modest increase in the coercive field (*E*_c_) with extended cycling, which suggests progressive domain wall pinning.

## 6 Conclusion

We fabricated highly crystalline MBI films on interdigital electrodes, which exhibit large spherulitic domains and near‐single‐crystal‐level remanent polarization. The polarization switching follows KAI kinetics with an Avrami index *n* ≈ 1 at low temperature and low field, consistent with quasi‐one‐dimensional switching along the hydrogen‐bond chains. With a stringent fatigue‐measurement protocol that ensures complete polarization reversal, the remanent polarization of MBI films remains close to the pristine value after 10^8^ continuous switching cycles over approximately 2 weeks. The robust endurance may arise from the combination of high crystallinity enabled by LDRC growth and the localized proton‐transfer switching mechanism in MBI, which introduces minimal structural distortion during polarization reversal. These results demonstrate that molecular ferroelectrics can achieve robust switching durability in interdigital architectures, highlighting hydrogen‐bonded systems such as MBI as promising platforms for reliable, low cost, and sustainable organic ferroelectric devices, while several limitations remain. The present endurance measurements were performed under a single high‐field driving condition and on interdigital geometries, which may differ from vertical device architectures used in practical applications. The generality of the results would also benefit from testing with alternative electrode materials beyond Pt to assess interface‐dependent effects. Finally, although the observed endurance likely benefits from localized proton‐transfer switching mechanism, the connection remains inferential, so direct structural or spectroscopic probes of proton dynamics during cycling will be needed to more conclusively establish the microscopic origin of the fatigue behavior.

## 7 Methods

The MBI films were deposited by thermal evaporation using a physical vapor deposition system (EvoVac, Angstrom Engineering) operated under high vacuum (~10^−6^ Torr). A schematic of the modified deposition setup is provided in our earlier work (Figure 1b) [20]. Film growth was carried out on glass substrates patterned with Pt IDEs (G‐IDEPT5, Metrohm, USA) featuring a 10 μm period and 4 μm electrode spacing (Figure S9a) [20]. The IDEs were interfaced with a Radiant Precision RT66C ferroelectric tester to perform electrical polarization measurements and switching dynamics analysis. Depositions were conducted at a substrate temperature of 230 K, regulated by a Lakeshore temperature controller employing liquid nitrogen circulation to achieve cryogenic conditions. Film thickness was monitored in situ using an integrated quartz crystal microbalance (QCM) and subsequently calibrated using several test samples, revealing a deviation of approximately 100 nm from the nominal thickness. The spherulitic morphology was examined using a reflective crosspolarized optical microscope (Nikon Eclipse L200N). Microscale surface topography was characterized with a Keyence laser scanning microscope, while nanoscale features were analyzed via atomic force microscopy (Bruker Icon) operated in ScanAssyst Peak Force Tapping mode. Structural characterization was performed using a Bruker D8 diffractometer equipped with a 2D detector.

## Funding

This study was funded by UNL Grand Challenges catalyst award entitled Quantum Approaches addressing Global Threats and Nebraska Center for Energy Sciences Research (NCESR).

## Conflicts of Interest

The authors declare no conflicts of interest.

## Supporting information

Supplementary Material

## Data Availability

The data that support the findings of this study are available from the corresponding author upon reasonable request.
