# Supplementary material for: Proton‐Transfer Ferroelectrics With High Switching Endurance
Source: Small Sci. 2026 Nov 5;6(11):e70438. doi: 10.1002/smsc.70438 (PMC13740487; doi:10.1002/smsc.70438)
Supplement: Supplementary file 1 [file SMSC-6-e70438-s001.pdf]

**Supplementary Figures:**

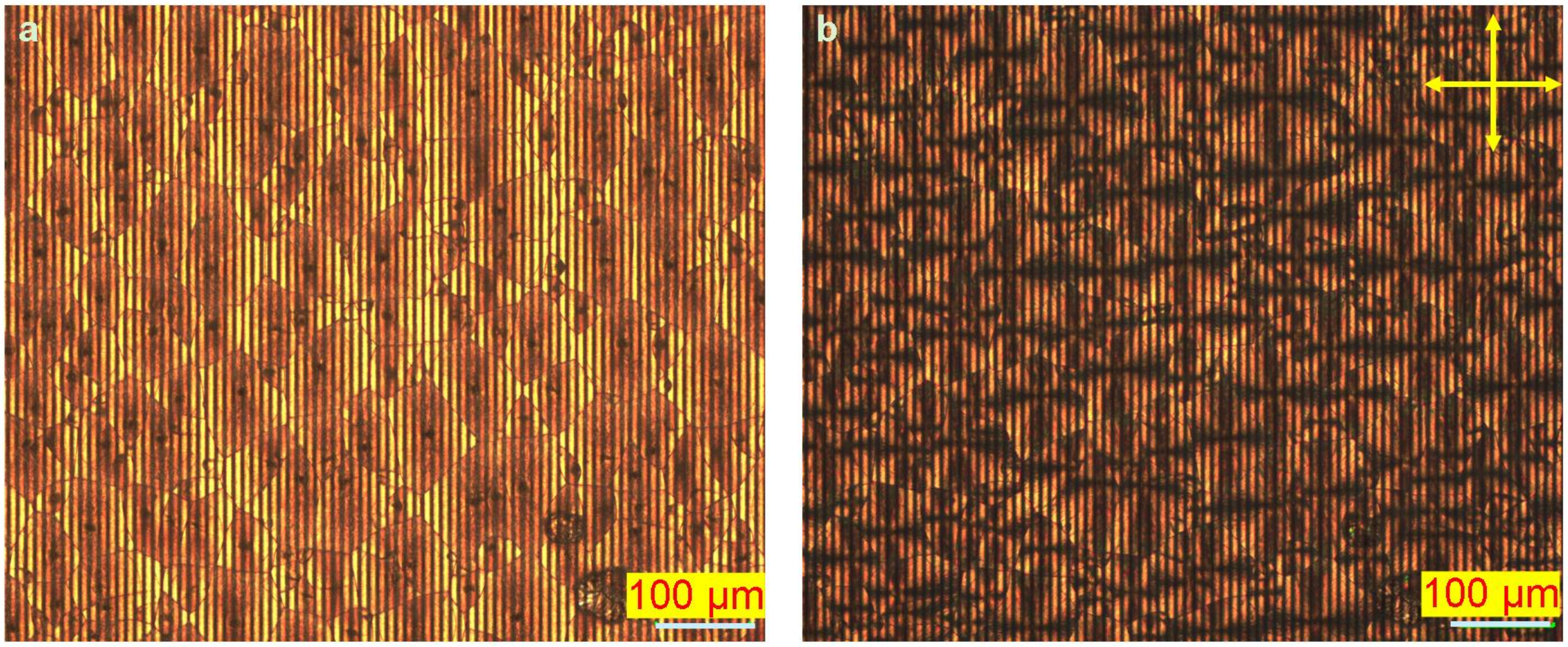


**Figure S1.** Optical Images of MBI grown on an IDE substrate. (a) and (b) shows the images in $0^0$ and $90^0$ (crossed position) between the polarizer and analyzer. The black spots on (a) are typical centers of the spherulitic morphology.

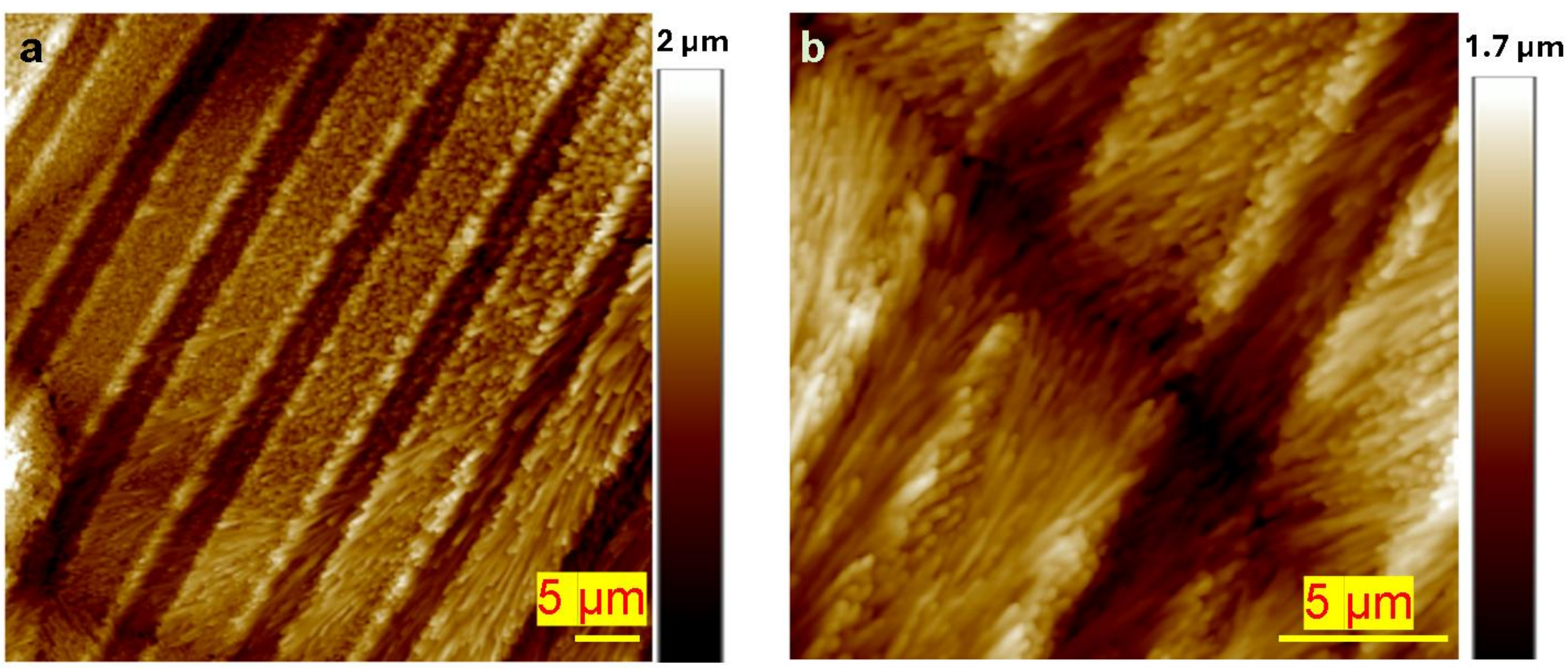


**Figure S2.** AFM images at different scales (a) 50 µm x 50 µm (b) 5 µm x 5 µm. RMS roughness is ~80 nm within one finger. The sample is 7 µm.

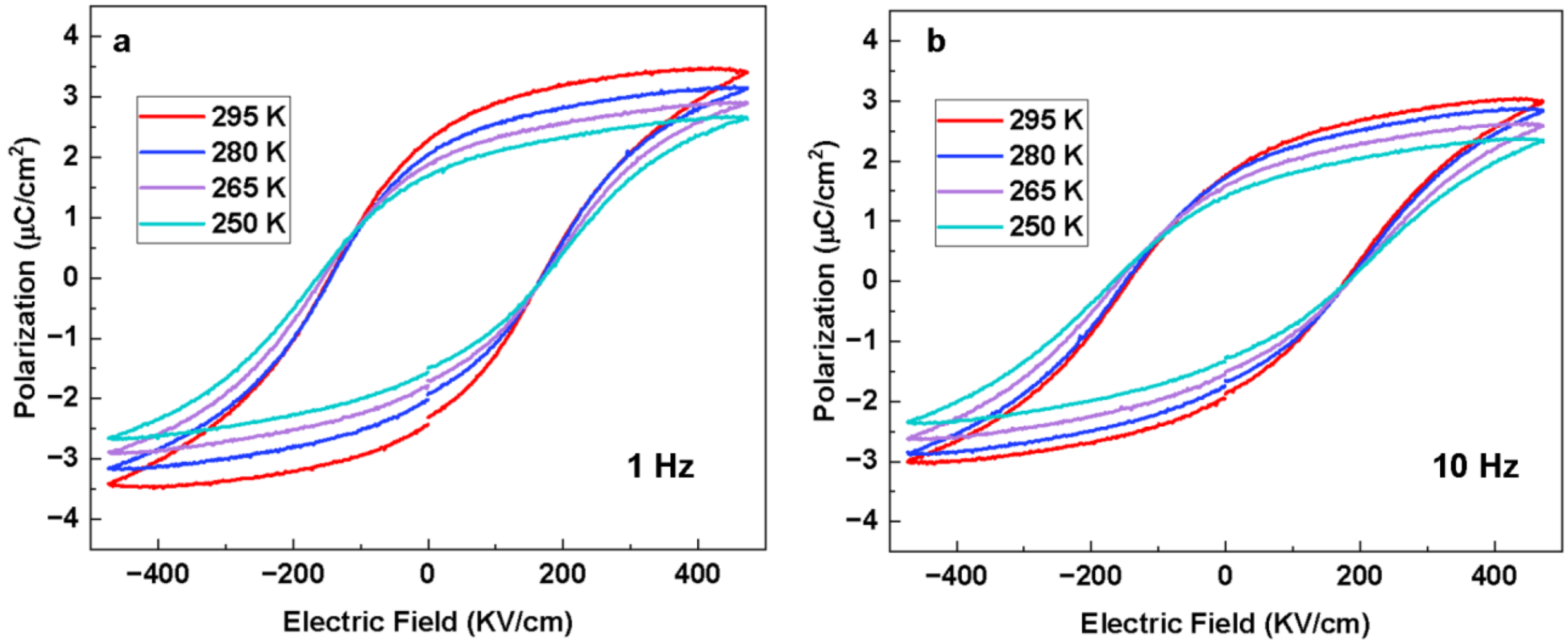


**Figure S3.** Polarization hysteresis loops of MBI at different temperatures at 1Hz (a) and 10 Hz (b) for 7 μm MBI sample.

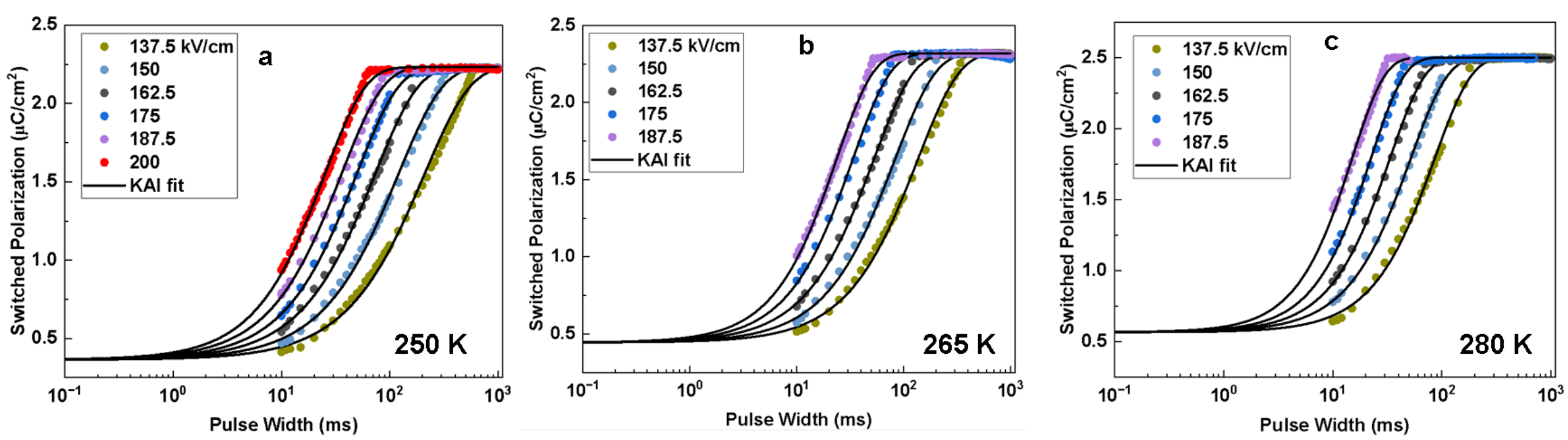


**Figure S4**. (a) Plot of switched polarization at several electric fields at temperatures: 250 K (a), 265 K (b) and 280 K (c) while the solid line is a fit to KAI model. The sample thickness is 7 μm.

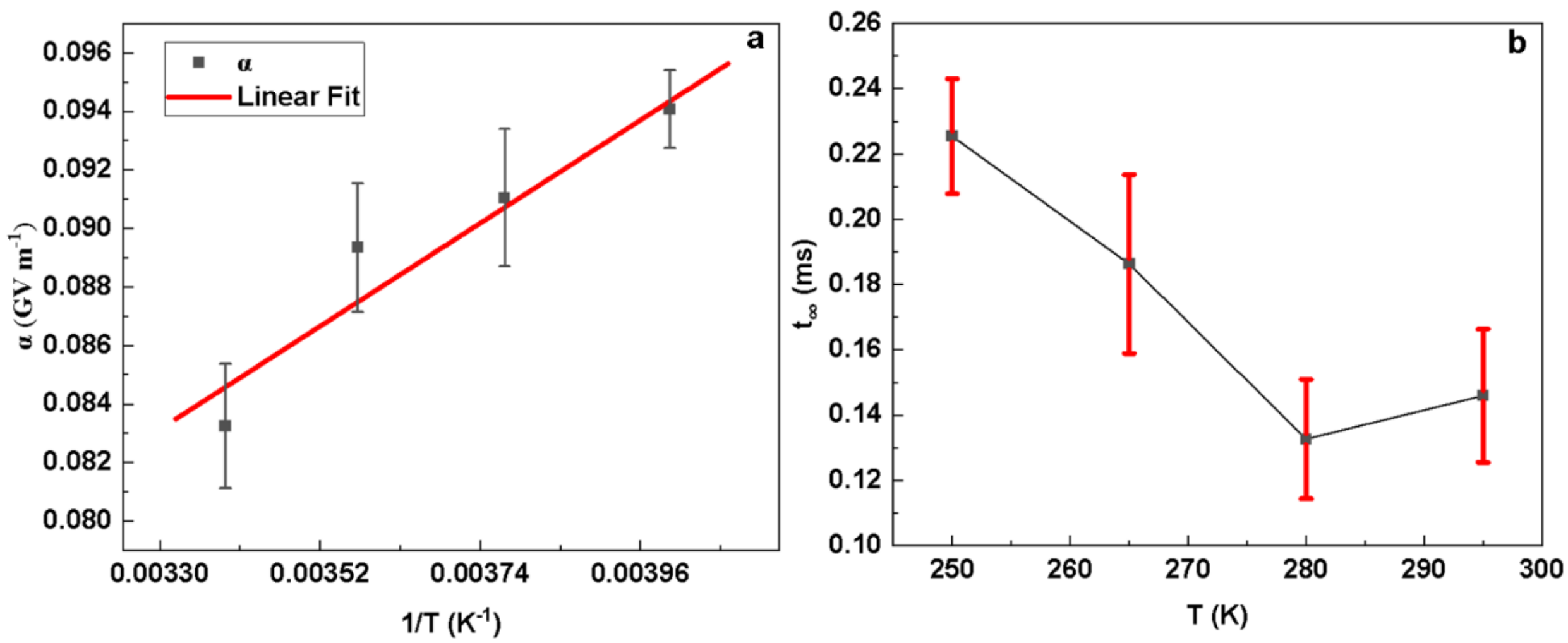


**Figure S5**. (a) Temperature dependence of the Merz law parameter α, plotted as a function of inverse temperature (1/T) with a linear fit. (b) shows the corresponding limiting switching time at infinite fields ($t_\infty$) as a function of temperature with dashed line being a guide to the eye.

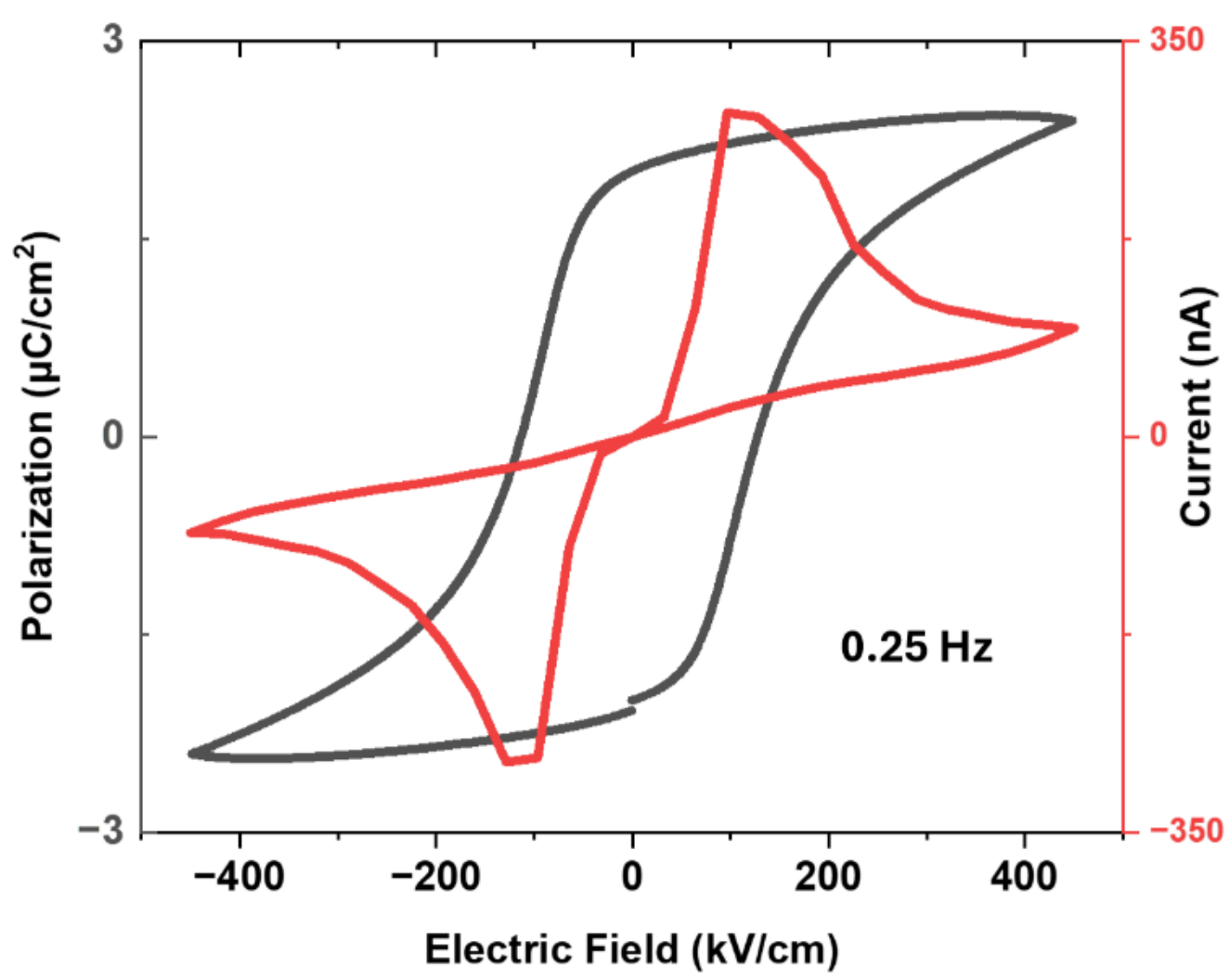


**Figure S6**. (a) Plot of Polarization Electric field (P-E) and Current Electric field (I-E) loop for 2 μm thick sample at 295 K at 0.25 Hz.

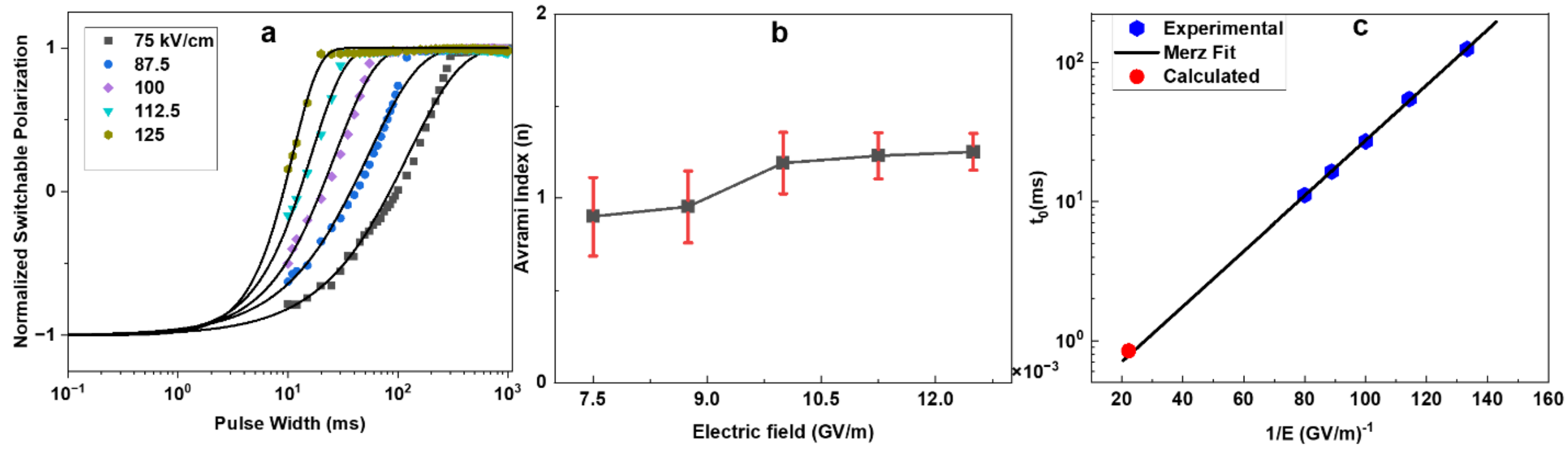


**Figure S7**. (a) Plot of normalized switchable polarization at several electric fields at room temperature (295 K) for 2 µm sample while the solid line is a fit to KAI model. (b) Plot of Avrami's index vs electric field at room temperature based on KAI model fit. (c) Plot of characteristic switching time vs inverse electric field from (a) while the solid line is a linearized Merz law fit. The calculated characteristic switching time at 180 V (450 KV/cm) is also shown as red solid circle.

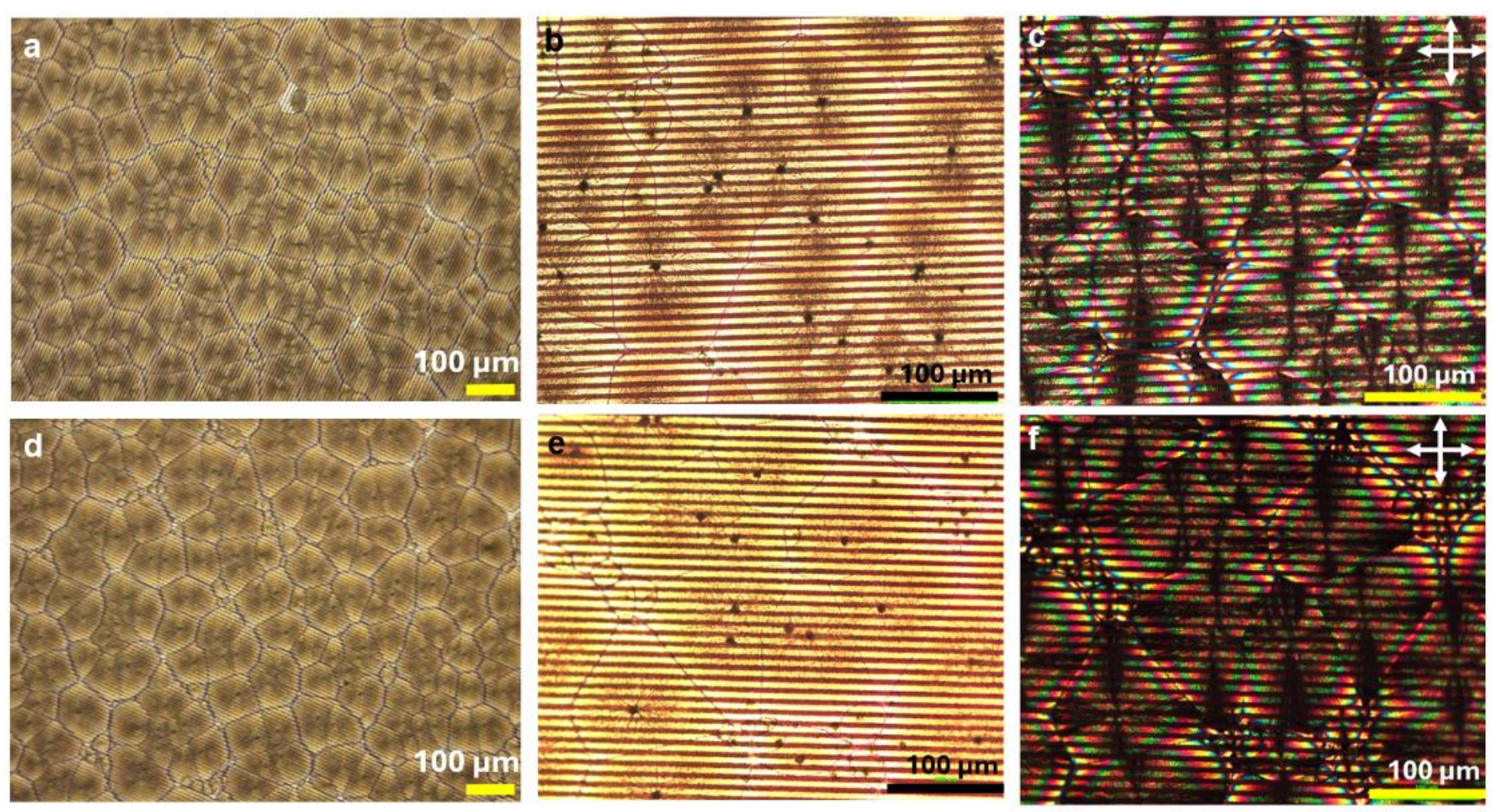


**Figure S8**. Top panels (a–c) show the pristine MBI film, while bottom panels (d–f) present the corresponding micrographs after ~$10^8$ stress cycles applied using an interdigital electrode configuration. Panels (a) and (d) display low-magnification optical images, whereas panels (b) and (e) show higher-magnification images acquired between crossed polarizers at 0°. Panels (c) and (f) correspond to the same regions observed under crossed polarizers rotated to 90°, highlighting the birefringent contrast associated with the film microstructure. No significant morphological degradation or visible changes in the optical domain structure are observed after electrical endurance cycling. The MBI film thickness is 2 µm.

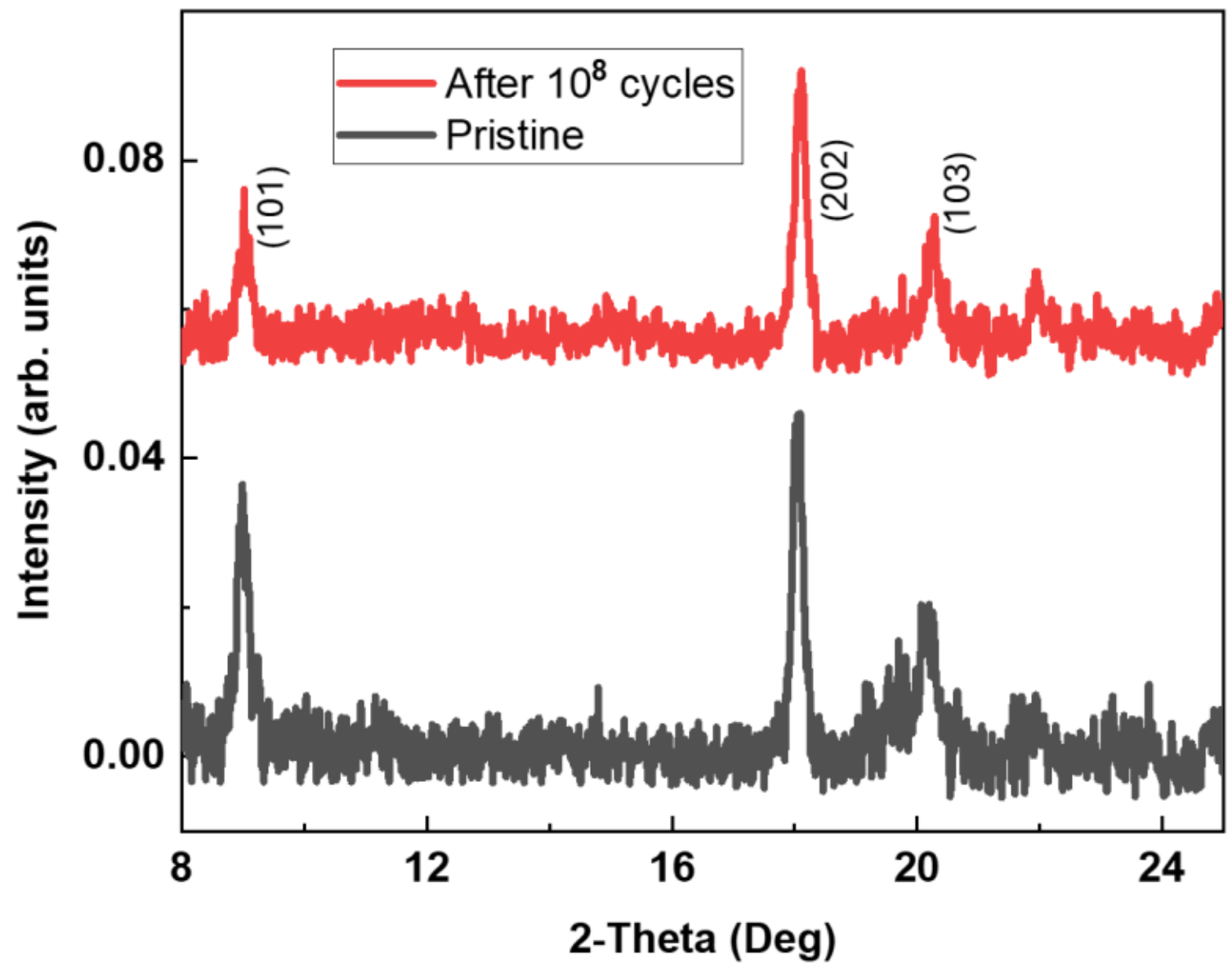


**Figure S9**. X-ray Diffraction for 2 μm MBI film: Pristine and after ~$10^8$ stress cycles.

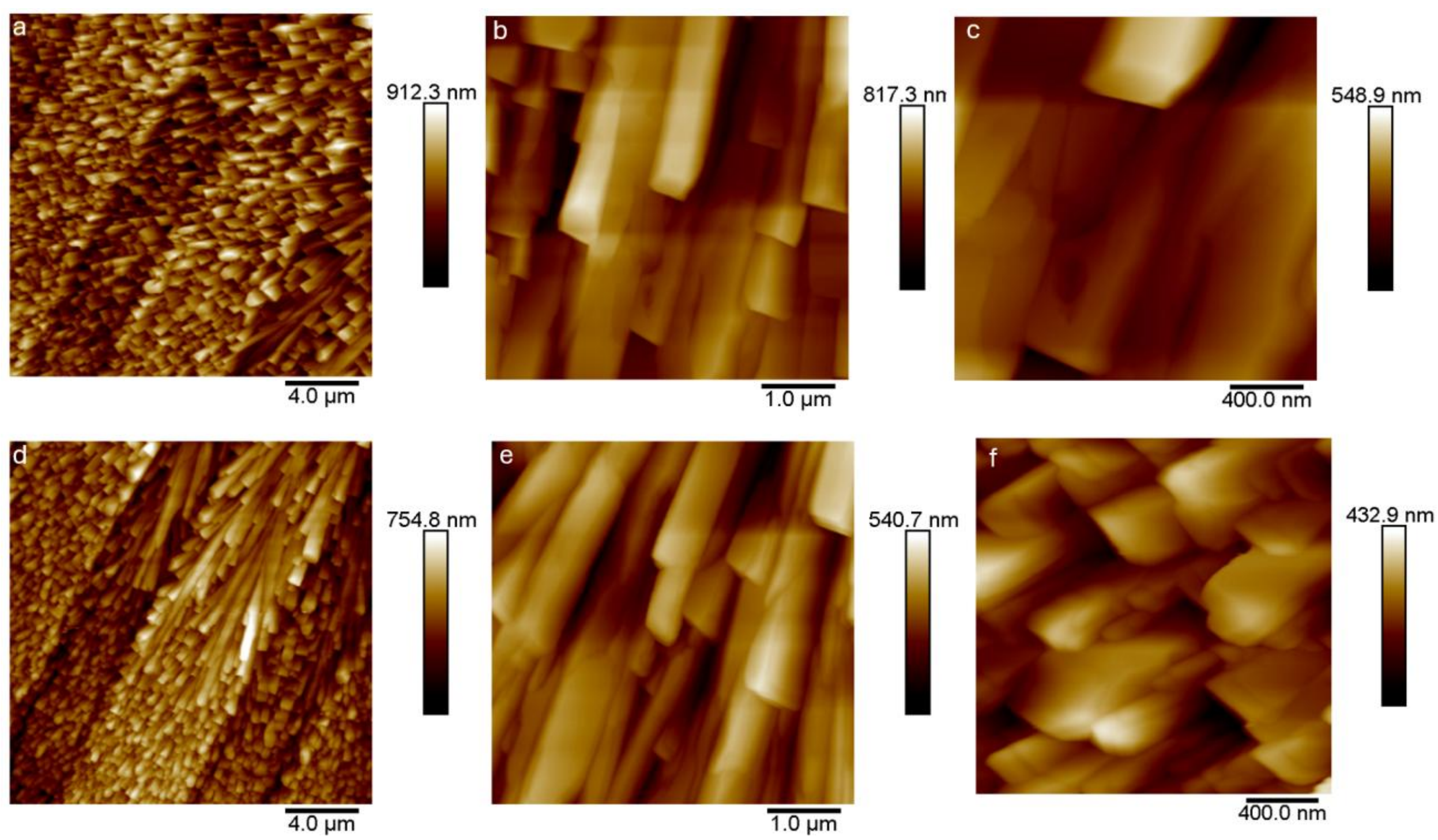


**Figure S10**. Panels (a), (b), and (c) present representative AFM images of the pristine MBI sample acquired at scan sizes of 20 μm × 20 μm, 5 μm × 5 μm, and 2 μm × 2 μm, respectively.

Panels (d), (e), and (f) show AFM images of the fatigued 2 μm-thick MBI film obtained using the same scan sizes for direct comparison.

**Note 1: Experimental protocol for fatigue stress measurements:**

Fatigue measurements were performed using the built-in long duration fatigue testing routine of the Radiant Precision RT66C ferroelectric tester. The sample was subjected to bipolar switching pulses with an amplitude of 180 V and pulse width $t_f = 5$ms. This pulse width is substantially larger than the characteristic switching time ($t_0$~0.84 ms) for the sample at the given voltage (electric field) based on switching dynamics measurement and extrapolated to 180 V (Fig. S7). This time is estimated from the linearized Merz law at lower electric fields as the direct measurement is beyond the tester capability at higher electric fields. After a specified number of fatigue cycles operated at 100 Hz frequency, the remanent polarization was evaluated using a standard built-in PUND measurement sequence in the tester. The PUND pulse amplitude was 180 V with a pulse width $t_p = 1000$ ms and delay of 500ms was chosen to relax any transients. This procedure was repeated throughout the fatigue experiment to monitor the polarization with cycling, and the corresponding Polarization-Voltage loops were measured in couple of decade intervals.

**Table S1**: Endurance performance and switching characteristics for selected ferroelectric systems, including PZT, P(VDF-TrFE), engineered polymer capacitors, and the present MBI molecular ferroelectric.

| **Material** | **Remanent Polarization (μC/cm$^2$)** | **Maximum Switching Fields applied (kV/cm)** | **Pulse Width/ Frequency** | **Operating Conditions** | **Endurance cycles** |
|---|---|---|---|---|---|
| Inorganic (PZT[1]) | ~27.3 | ~65 nm, 500 kV/cm | 100 kHz, bipolar square | Room Temperature (RT) | >$10^{10}$ cycles, stable |
| P(VDF-TrFE[2]) | ~7 | ~500nm, 600 kV/cm | 100 Hz, bipolar triangular | RT | $10^5$ (~50 % reduction in Pr) |
| Engineered P(VDF-TrFE) capacitor[3] | ~7 | ~150 nm, 1530 kV/cm | 100 kHz, Pulses | RT | $10^7$ (~64 % reduction in Pr) |
| MBI (this work) | ~3 | ~2 μm, 450 kV/cm | 100 Hz, Pulses | RT | $10^8$ (~15 % reduction in Pr |

| | | | | | |
|---|---|---|---|---|---|
| | | | | | reference to post wake-up peak) |

**Table S2**: Activation barrier for some of the ferroelectric systems

| **System** | **Activation barrier (GVm$^{-1}$)** |
|---|---|
| Inorganic Ferroelectrics: BaTiO3 and PZT[4–7] | 0.3-1 |
| Ferroelectric Polymers (PVDF,P(VDF-TrFE))[8–11] | 0.1-0.3 |
| MBI (this work) | ~0.08 |

**Table S3**: Fitting parameters from the KAI model for the 2 μm sample.

| **Electric Field (kV/cm)** | **Avrami's Index (n)** | **Characteristic Switching Time (ms)** |
|---|---|---|
| 75 | 0.9 | 125.56 |
| 87.5 | 0.95 | 54.56 |
| 100 | 1.19 | 27.06 |
| 112.5 | 1.23 | 16.42 |
| 125 | 1.25 | 11.11 |

References:


1. Maderic, BP and Sanchez, LE and Wu, S. Ferroelectric switching, memory retention and endurance properties of very thin PZT films. *Ferroelectrics* **116**, 37–41 (1991).

2. Zhao, D. *et al.* Polarization fatigue of organic ferroelectric capacitors. *Sci. Rep.* **4**, 1–7 (2014).

3. Wu, J. *et al.* Improvement of endurance and compatibility of organic ferroelectric polymer through inserting Al2O3 interfacial layer. *Phys. Rev. B* **214111**, 1–8 (2025).

4. Jo, J. Y. *et al.* Domain switching kinetics in disordered ferroelectric thin films. *Phys. Rev. Lett.* **99**, 1–4 (2007).

5. Scott, J. F. Nanoferroelectrics: Statics and dynamics. *J. Phys. Condens. Matter* **18**, (2006).

6. Merz, W. J. Switching time in ferroelectric BaTiO3 and its dependence on crystal thickness. *J. Appl. Phys.* **27**, 938–943 (1956).

7. Zhukov, S., Genenko, Y. A. & von Seggern, H. Experimental and theoretical investigation on polarization reversal in unfatigued lead-zirconate-titanate ceramic. *J. Appl. Phys.* **108**, (2010).

8. Katsouras, I. *et al.* The negative piezoelectric effect of the ferroelectric polymer poly(vinylidene fluoride). *Nat. Mater.* **15**, 78–84 (2016).

9. Hu, W. J. *et al.* Universal ferroelectric switching dynamics of vinylidene fluoride-trifluoroethylene copolymer films. *Sci. Rep.* **4**, 1–8 (2014).

10. Cornelissen, T. D., Cornelissen, T. D., Urbanaviciute, I., Kemerink, M. & Kemerink, M. Microscopic model for switching kinetics in organic ferroelectrics following the Merz law. *Phys. Rev. B* **101**, (2020).

11. Cornelissen, T. D., Cornelissen, T. D., Urbanaviciute, I., Kemerink, M. & Kemerink, M. Microscopic model for switching kinetics in organic ferroelectrics following the Merz law. *Phys. Rev. B* **101**, 214301 (2020).
